# Brief research report: in-depth immunophenotyping reveals stability of CD19 CAR T-cells over time

**DOI:** 10.3389/fimmu.2024.1298598

**Published:** 2024-01-22

**Authors:** Ivan Odak, Lâle M. Bayir, Lennart Riemann, Ruth Sikora, Jessica Schneider, Yankai Xiao, Nora Möhn, Thomas Skripuletz, Gernot Beutel, Matthias Eder, Arnold Ganser, Reinhold Förster, Christian R. Schultze-Florey, Christian Koenecke

**Affiliations:** ^1^ Institute of Immunology, Hannover Medical School, Hannover, Germany; ^2^ Department of Hematology, Hemostasis, Oncology and Stem Cell Transplantation, Hannover Medical School, Hannover, Germany; ^3^ Department of Pediatric Pneumology, Allergology and Neonatology, Hannover Medical School, Hannover, Germany; ^4^ Department of Neurology, Hannover Medical School, Hannover, Germany

**Keywords:** CAR T-cell, DLBCL diffuse large B-cell lymphoma, ALL acute lymphoblastic leukemia, tisagenlecleucel tisa-cel, spectral flow cytometry, immunophenotyping, CRS cytokine release syndrome, ICANS immune effector cell associated neurotoxicity syndrome

## Abstract

Variability or stability might have an impact on treatment success and toxicity of CD19 CAR T-cells. We conducted a prospective observational study of 12 patients treated with Tisagenlecleucel for CD19^+^ B-cell malignancies. Using a 31-color spectral flow cytometry panel, we analyzed differentiation stages and exhaustion markers of CAR T-cell subsets prior to CAR T-cell infusion and longitudinally during 6 months of follow-up. The majority of activation markers on CAR T-cells showed stable expression patterns over time and were not associated with response to therapy or toxicity. Unsupervised cluster analysis revealed an immune signature of CAR T-cell products associated with the development of immune cell-associated neurotoxicity syndrome. Warranting validation in an independent patient cohort, in-depth phenotyping of CAR T-cell products as well as longitudinal monitoring post cell transfer might become a valuable tool to increase efficacy and safety of CAR T-cell therapy.

## 1 Introduction

CD19-directed chimeric antigen receptor (CAR) T-cell therapy has become a standard-of-care treatment for patients with relapsed or refractory diffuse large B-cell lymphoma (DLBCL) and B-cell lineage acute lymphoblastic leukemia (B-ALL). For both entities the CD19 CAR T-cell product Tisagenlecleucel was approved for demonstrating curative potential (1, 2), which was also confirmed in real-world analyses (3). However, this treatment harbors potentially life-threatening side effects such as cytokine release syndrome (CRS) and immune effector cell-associated neurotoxicity syndrome (ICANS), which require meticulous patient observation and trained medical staff to provide adequate clinical management (4). The incidence of CRS after treatment with Tisagenlecleucel has been reported to range between 57-93% (1, 5, 6), with severe CRS (≥ grade 3) (7) emerging in about 10-30% of patients (3, 8, 9). ICANS has been described to occur in about 20-70% of patients treated (10) and severe neurotoxicity (≥ grade 3) manifested in 10-35% of patients respectively (3, 11, 12). Although isolated ICANS can occur, manifestation of severe CRS has been associated with a higher risk of concomitant neurotoxicity (13). Risk factors for CRS and ICANS have been described (13–19), but specificity and sensitivity for these broad predictive markers appear to be low.

To date, cell intrinsic factors determining the outcome and side effects of CAR T-cell therapy are largely unknown. The CAR T-cell immune phenotype before and after cell transfer may become a suitable and readily available biomarker to predict outcome and toxicity. A recent study described CAR T-cells with higher expression of co-inhibitory molecules (LAG3 and PD1) as well as lower expression of the cytotoxicity marker CD107a to be associated with favorable outcome (19). Common use of methods such as Time-of-Flight Cytometry or spectral flow cytometry allows almost unparalleled resolution in terms of cell phenotyping on a single-cell protein level. Given the vast amount of data obtained by these methods, an analysis pipeline is needed to decipher it in a meaningful way.

In this study, we prospectively analyzed the CAR T-cell immune phenotype kinetics in the context of patients’ clinical course. To this end, we applied a 31-color spectral flow cytometry approach to identify differentiation stages and exhaustion status of the CAR T-cell product itself and longitudinally in patients’ blood samples during 6 months of follow-up. In addition to a conventional gating approach, we employed a quantitative unsupervised clustering analysis. Ultimately, we provide data on the dynamics of expression of various T-cell markers over time and their correlations with response to therapy and occurrence of CRS or ICANS.

## 2 Materials and methods

### 2.1 Cohort and study design

Between July 2019 and May 2020 we recruited 16 patients diagnosed with relapsed/refractory DLBCL or relapsed/refractory ALL, who received treatment with Tisagenlecleucel in the Department of Hematology, Hemostasis, Oncology and Stem Cell Transplantation at Hannover Medical School (Germany). We obtained written, informed consent from all participants. As shown in **Supplementary Figure S1**, we excluded four patients from the analysis: two patients due to lack of biomaterials, one patient received a cell product that did not comply with the manufacturer’s standard regarding minimal cell count and in one case the sample quality of the obtained biomaterial was insufficient for further analysis. This study was designed in accordance with the Declaration of Helsinki and approved by the institutional review board of Hannover Medical School (8610 _BO_K_2019). The study layout is shown in **Supplementary Figure S2**.

Tisagenlecleucel was administered in an inpatient setting. The cell product bag was flushed with NaCl 0.9% to administer as many CAR T-cells as possible to the patient. Prior to disposal, the cell product bag was flushed again and left-over cells were used for analysis in flow cytometry. A standardized follow-up, consisting of a minimum of 10 days of hospitalization and subsequent scheduled outpatient appointments, was implemented (**Supplementary Figure S1**). Thorough neurological screening was performed by specialists to monitor occurrence of respective side effects (11). Response to therapy was determined 30 days as well as 90 days after CAR T-cell infusion. In most cases, response evaluation was based on PET-CT scans with response assessment according to Lugano classification (20). In case of unavailability of PET-CT scan, conventional CT staging was performed.

### 2.2 Flow cytometry

**Figure 1:**
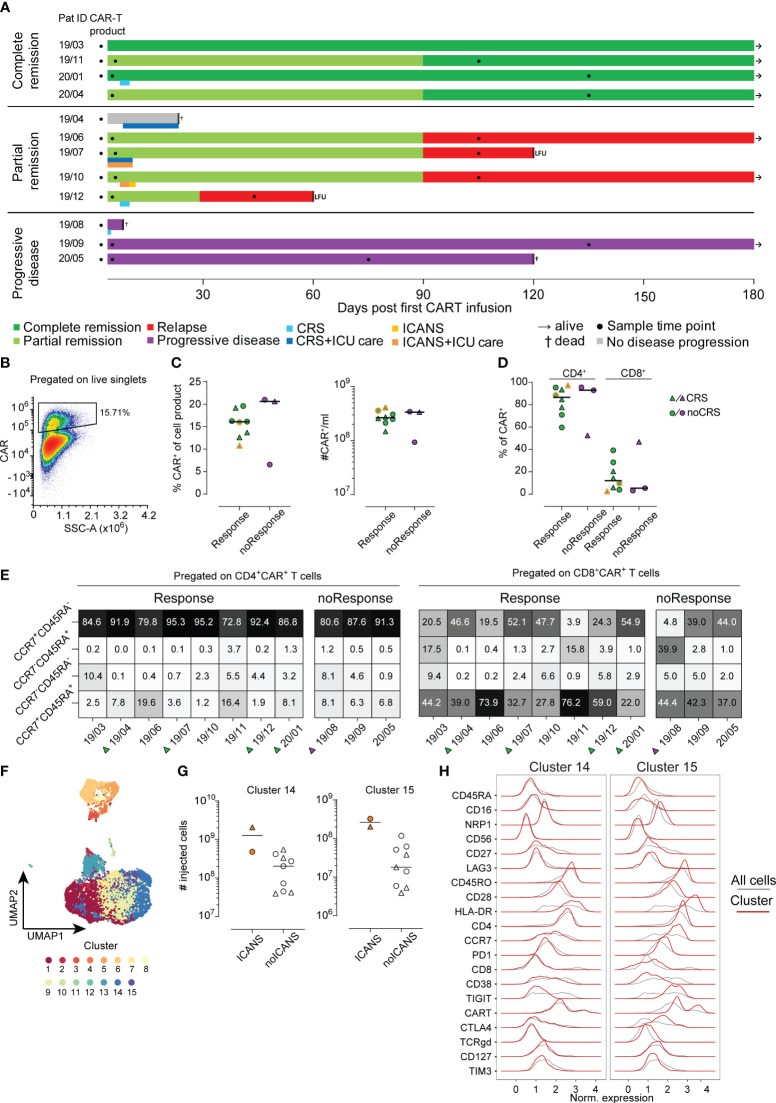
Analysis of CAR T-cell product. **(A)** Each patient is represented by one line with color coding response to CAR T-cell therapy (dark green complete remission, light green partial remission, red relapse, grey no evidence of disease progression, purple progressive disease). A second color-coded line below the response indicates occurrence of CRS (blue) and ICANS (orange). Sampling is indicated by filled black circles. Follow-up time was 180 days. LFU indicates loss to follow-up. **(B)** Example for CAR^+^ T-cell gating **(C)** Percentages (left) and absolute cell numbers (right) of infused CAR^+^ T-cells is shown. Lines represent median. **(D)** Distribution of CD4^+^ and CD8^+^ CAR^+^ cells in the CAR T-cell product. **(E)** Distribution of CAR^+^ subpopulation based on their CCR7 and CD45RA expression pattern. **(F)** UMAP visualization identifying 15 distinct cell clusters in CAR^+^ T-cells of the cell product (n=11). **(G)** Numbers of injected cells with cluster 14 (left) or cluster 15 (right) phenotype in the CAR T-cell products of patients with or without ICANS development. **(H)** Detailed surface marker phenotype of cells within clusters 14 and 15. Red line shows expression of cells of the indicated cluster only, grey line indicates expression of all cells.

Within this study, we obtained whole blood samples from patients to process into peripheral blood mononuclear cells (PBMCs) using Ficoll gradient centrifugation as described elsewhere (21), which were cryopreserved at -80°C. We set up a custom-built antibody panel to discriminate T-cell subsets and investigate surface expression of activation and exhaustion markers (**Supplementary Table S1**). The range of analyzed cells in the CAR T-cell gate for the CAR T-cell products was 219-7482 with the median being at 3793. For the patient’s samples, the range of analyzed cells in the CAR T-cell gate was 128-11354 with the median being 968. Samples were stained at room temperature, using 30 monoclonal antibodies and a viability dye, and washed twice. We used an Aurora spectral flow cytometer (Cytek) equipped with five lasers (355 nm, 405 nm, 488 nm, 561 nm, 640 nm) to acquire the primary data. Data analysis was performed using SpectroFlo version 2.2.0 (Cytek) and FCS Express™ 7 (Denovo). Gating of CAR^+^ cells is shown in **Figure 1B**.

### 2.3 Unsupervised quantitative cluster analysis

First, conventional 2D gating was performed to remove dead cells and doublets for data clean-up. Additionally, for patient samples, non-leukocyte cells (CD45RA^-^CD45RO^-^) and monocytes (CD14^+^) were also removed prior to data export. We then proceeded to further investigate the samples using an unsupervised clustering approach (**Figure 1F**). Transformation of data was performed via the Logicle function of the R FlowCore package (version 2.2.0, Bioconductor) (22). In the next step, the FlowAI algorithm (version 1.20.1, Bioconductor) was applied for quality control (23). The FlowSOM algorithm (version 1.22.0, Bioconductor) was used for clustering the data according to default settings (24). The number of FlowSOM meta-clusters was set to 15 for the CAR T-cell products and 20 for analysis of all cells. Ultimately, the Uniform Manifold Approximation and Projection (UMAP, **Figure 1F**) approach served for dimensionality reduction. Before clustering, expression of all markers was checked manually for each sample using histogram plots. In case of absence of expression dynamics (equal in all samples) the respective marker was excluded from the analysis to avoid technical artefacts. Manual cluster annotation was set up considering relative expression of markers among clusters.

### 2.4 Statistical analysis

We used Prism 7 (GraphPad) for statistical analysis. All data was tested for normality of distribution and tested with appropriate test as indicated in the figure legends. For comparison of mean fluorescence intensity (MFI) values of various activation and exhaustion molecules expressed on CAR^+^ T-cells within the cell product and at early and late time points two way ANOVA with Tukey’s *post hoc* test was used.

## 3 Results

A total of 16 patients were recruited to this prospective observational study, of whom 12 met the inclusion criteria (**Supplementary Figure S1**). Eight of 12 patients had a complete (CR, N=4) or partial remission (PR, N=4) at three months post CAR T-cell therapy with a median duration of response of 13.5 months (range 8-24) (**Figure 1A**). Three patients did not respond to CAR T-cell therapy and showed progressive disease (PD, **Figure 1A**). In the subset of patients who initially experienced PR, one patient progressed one month after CAR T-cell infusion and three subjects showed relapsed disease at three months of follow-up. One patient died from neutropenic sepsis 23 days post CAR T-cell infusion without signs of disease progression. Detailed clinical characteristics are shown in **Table 1** and **Supplementary Tables S2**, **S5**.

**Table 1:** Patients’ characteristics prior to CART therapy.

Pat ID	Age	Sex	Disease	Molecular rearrange-ments	Ann Arbor Stage	Elevated LDH	Extra-nodal manifes-tations	CNS manifes-tation	ECOG	IPI	Previous lines of antineo-plastic therapy	Previous SCT	Disease status prior to CART infusion	Standard lympho-depletion regimen	Bridging CTx	Bridging RTx	Days from apheresis to CART infusion
**19/03**	60	M	tFL	–	IVAE	No	Yes	No	1	3	3	No	PD	Yes	No	No	54
**19/04**	64	M	DLBCL	BCL2+	IVAEX	Yes	Yes	No	1	4	3	Autologous	PD	Dose red. (Renal insufficiency)	No	No	62
**19/06**	31	F	DLBCL	BCL2+, BCL6+ C-MYC+	IIAE	No	Yes	No	2	1	2	Autologous	PD	Yes	Yes	Yes	196
**19/07**	74	F	DLBCL	–	IIIA	No	No	No	1	2	4	Autologous	PD	Yes	No	Yes	89
**19/08**	36	M	tFL	BCL2+, C-MYC+	IVBEX	Yes	Yes	No	0	2	3	No	PD	Yes	Yes	Yes	56
**19/09**	59	F	DLBCL	BCL2+ BCL6+	IIIBE	Yes	Yes	No	1	2	3	Autologous	PD	Yes	No	No	38
**19/10**	65	M	tFL	BCL2+ BCL6+	IVBE	No	Yes	No	1	3	2	No	PD	Dose red. (Renal insufficiency)	No	No	70
**19/11**	66	M	DLBCL	BCL2+	IVAE	No	Yes	No	1	2	3	Autologous	PD	Yes	No	No	77
**19/12**	32	M	DLBCL	BCL2+ BCL6+ C-MYC+	IIAE	No	Yes	No	1	1	2	No	PD	Yes	No	No	35
**20/01**	22	M	cALL	NA	/	No	/	Yes	0	/	8	Allogeneic	PD	Yes	No	Yes	115
**20/04**	56	F	DLBCL	BCL2+ BCL6+	IIIAE	Yes	Yes	No	2	1	2	Autologous	PD	Yes	No	Yes	54
**20/05**	75	M	DLBCL	BCL6+	IVAE	Yes	Yes	No	1	1	4	No	PD	Yes	No	Yes	60

Pat ID, Patient ID; LDH, Lactate dehydrogenase; CNS, Central nervous system; ECOG, Eastern Cooperative Oncology Group Status; IPI, International Prognostic Index; SCT, Stem cell transplantation; CART, Chimeric antigen receptor T-cell; CTx, Chemotherapy; RTx, Radiotherapy; tFL, Transformed follicular lymphoma; DLBCL, Diffuse large B-cell lymphoma; cALL, Common acute lymphoblastic leukemia; NA, Not applicable; PD, Progressive disease; Dose red, Dose reduced.

CRS manifested in five out of 12 patients (41.7%), of whom 3 cases were classified as °I and could be managed with supportive care only (7). One patient developed CRS °IV and later met diagnostic criteria for secondary hemophagocytic lymphohistiocytosis; he died from neutropenic sepsis. One patient had CRS °II and concomitant ICANS °II, which was treated with tocilizumab and dexamethasone. Another patient suffered from ICANS °II and received dexamethasone alone (**Supplementary Table S4**).

### 3.1 In depth phenotyping of CAR T-cell products does not reveal differences with regard to outcome upon conventional 2D analysis

**Figure 2:**
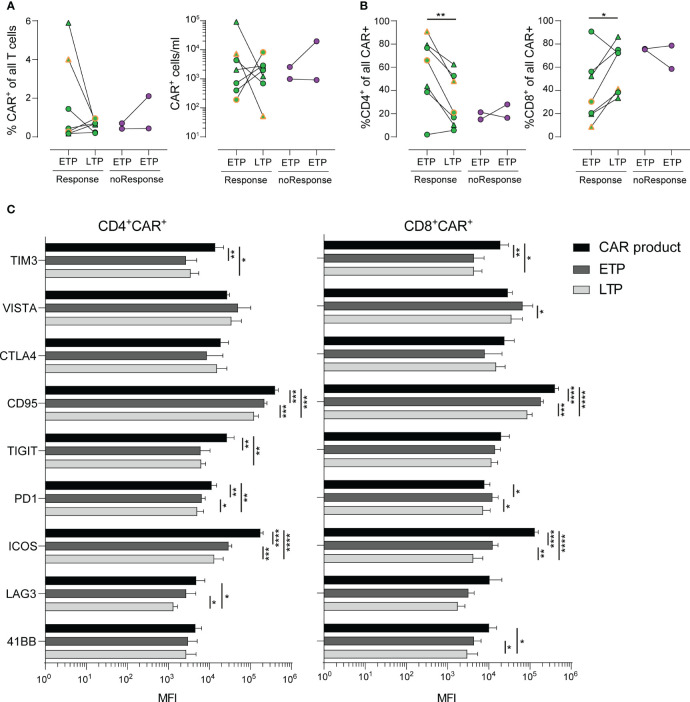
Stability and long-term phenotype of CAR^+^ T-cells. **(A)** Frequencies (left) and numbers (right) of CAR^+^ T-cells in PBMCs of patients in early (ETP) and late (LTP) time points post CAR T-cell infusion. **(B)** Distribution of CD4^+^ (left) and CD8^+^ (right) CAR^+^ cells of all CAR^+^ cells. **(C)** Mean fluorescence intensity (MFI) values of various activation and exhaustion molecules expressed on CAR^+^ T-cells within the cell product (black) and at early (dark grey) and late (light grey) time points. Lines represent mean. Statistics was done by two-way paired t test **(A, B)** or two way ANOVA with Tukey’s *post hoc* test **(C)**. *p<0.05; **p<0.01; ***p<0.001; ****p<0.0001.

We analyzed the phenotype of the infused CAR T-cell products (n=11) using conventional 2D gating. In order to assess differences with regard to outcome after CAR T-cell therapy, we separated the cohort based on response criteria into responders (n=8) and non-responders (n=3). Moreover, patients with CRS and ICANS were highlighted throughout the figures when applicable (**Figures 1C–E**, **2A, B**). No differences were detected when comparing the percentage and absolute counts of CAR T-cells within the CAR T-cell product for responders versus non-responders (**Figure 1C**). Notably, the two patients with ICANS had received the highest absolute count of CAR^+^ T-cells within the cohort (**Figure 1C**, indicated by dual-coloring). However, we did not observe any differences between the groups based on the occurrence of CRS (**Figure 1C**).

Of note, CD4^+^ CAR^+^ T-cells made up around 80% of the whole CAR T-cell product in this cohort. However, we did not detect an association of CD4/CD8 distribution in respect to treatment success or side effects (**Figure 1D**).

Next, we analyzed the CAR T-cells before infusion based on their CCR7 and CD45RA expression pattern. The combination of CCR7 and CD45RA expression is often used to describe naïve (CCR7^+^CD45RA^+^), effector/memory (CCR7^-^CD45RA^-^), TEMRA (CCR7^-^CD45RA^+^) and central memory (CCR7^+^CD45RA^-^) T-cells (25). In our cohort, the great majority of both CD4^+^ and CD8^+^ CAR T-cells expressed CCR7 and/or CD45RA (**Figure 1E**). CCR7 expression indicates the ability of CAR T-cells to enter the secondary lymphoid organs. After all, we could not detect a significant association of the expression of CCR7 and CD45RA with anti-tumor activity or toxicity (**Figure 1E**). In conclusion, conventional 2D analysis of the CAR^+^ T-cell population of the CAR T-cell products did not reveal any statistically significant differences regarding quantity or activation phenotype neither in responders versus non-responders nor in patients with or without CRS and/or ICANS.

### 3.2 Unsupervised cluster analysis identifies patients developing ICANS

Beyond conventional gating, we performed an unsupervised cluster analysis (26) of the infused CAR T-cell product (n=11) as well as across all available samples (n=29). As described in the Methods section cells were separated into 15 distinct clusters for the analysis of the infused CAR T-cell products (**Figure 1F**) and 20 distinct clusters in the case of all samples analysis (**Supplementary Figure S2A**). Due to lack of statistical power, we did not focus on correlations of clinical outcome with cell clusters but used a descriptive data analysis approach. Interestingly, the two patients developing ICANS received CAR T-cell products with a higher amount of cells with cluster 14 and 15 phenotype (**Figure 1G**). A closer look into the phenotype of cluster 14 and 15 revealed a shared similarity in most of the molecules. Yet, cluster 14 was defined by lack of expression of CCR7, PD1, TIGIT and CTLA4 compared to cluster 15. Both of these cell clusters were made up of CD4^+^ T-cells, however cluster 15 also had a higher proportion of CAR^+^ T-cells compared to cluster 14 (**Figure 1H**). In order to evaluate possible interplay of other cell types with CAR T-cells, we performed an integrated analysis of all samples from the CAR T-cell products, ETP and LTP (n=29). This analysis yielded 20 unique clusters (**Supplementary Figure S2A**). While we did observe some longitudinal differences, due to our small sample size, we did not identify any pattern that would separate responders from non-responders or CRS and/or ICANS patients (**Supplementary Figure S2B**). Still, a closer analysis identified CD4^+^CAR T and CD8^+^CAR T-cells predominantly making up clusters 5 and 12 respectively, but also present in clusters 4, 8, 9 and 11 (**Supplementary Figures S3A, B**).

### 3.3 The majority of activation markers on CAR^+^ T-cells show stable expression over time

Subsequently, we focused on the CAR T-cell kinetics within the patients’ peripheral blood at early time points (ETP, n=9, median 2 days, range 2-3 days) and late time points (LTP, n=9, median 105 days, range 48-130 days) post CAR T-cell infusion (**Figure 1A**; **Supplementary Figure S1A**).

As shown in **Figure 2A**, the percentage of CAR T-cells within the patients’ blood as well as the absolute count of CAR T-cells showed a heterogeneous dynamic, with some patients exhibiting a steep decline, while others had stable or expanding CAR T-cell populations. Of note, we observed increased frequencies of CAR T-cells with a CD8^+^ phenotype in patients with response by day 30 and a paralleled drop in frequencies of CD4^+^ CAR T-cells (**Figure 2B**).

Despite our deep phenotyping, we failed to observe differential expression of any one activation or checkpoint molecule prior to infusion that might be associated with development of either response to treatment or occurrence of CRS or ICANS. Finally, we analyzed the expression kinetics of activation markers, such as TIM3, VISTA, CTLA4, CD95, TIGT, PD1, ICOS, LAG3 and 4-1BB in a longitudinal time line. CD4^+^ and CD8^+^ CAR T-cells exhibited a similar expression kinetic of the majority of markers related to T cell exhaustion (**Figure 2C**). Notably, despite the obvious heterogeneity in terms of treatment of each patient, we observed a marked decrease of expression of TIM3, CD95, ICOS and TIGIT (in CD4^+^ CAR T-cells only) during the late time points throughout the cohort. Taken together, the detailed description of expression kinetics sheds light on the underlying complexity of CAR T-induced immune responses, including tumor escape mechanisms (27).

## 4 Discussion

Our approach of in-depth phenotyping of T-cell subsets of the CAR T-cell product as well as patient samples early and late after CAR T-cell therapy in a small cohort focused primarily on identification of differential expression of surface molecules as a function of outcome and toxicity.

Upon conventional 2D analysis of the CAR^+^ T-cell population of the CAR T-cell products we did not observe statistically significant differences regarding quantity or activation phenotype associated with response nor toxicity. While a clear causative relationship between dose of transferred CAR T-cells and occurrence of CRS or ICANS has not yet been demonstrated, several groups have identified high numbers of transferred CAR^+^ T-cells or increased expansion as a risk factor (13, 19, 28, 29). It is worth noting, that following lentiviral transduction and expansion protocols used in creation of the cell product, CCR7^+^CD45RA^+^ CAR T-cells likely do not have the same functional capacity as compared to non-genetically modified naïve T-cells (30). In our cohort, the great majority of both CD4^+^ and CD8^+^ CAR T-cells expressed CCR7, thus likely retaining their ability to enter lymph nodes which represents an important aspect of their potency to clear lymphatic tumors (31, 32).

In contrast to the standard 2D gating approach, employing an unsupervised cluster analysis of the CAR T-cell products we separated 15 distinct clusters, which were analyzed in a descriptive manner. Two clusters were associated with ICANS. Both clusters 14 and 15 contained CAR^+^ T-cells and the overall exhausted-like phenotype of these two clusters resembled each other. Besides the amount of CAR^+^ T-cells, they diverged only in expression of CCR7, PD1, TIGIT and CTLA4. While the pivotal role of CCR7 has been described above, both TIGIT and CTLA4 are immune checkpoints that regulate immune function and have been shown to influence anti-tumor control (33–35). Cluster 15, which had a higher proportion of CAR^+^ T-cells, simultaneously exhibited a higher expression of TIGIT and CTLA4, implying a more activated state. Considering that patient ID 19/07 had both CRS and ICANS we cannot rule out that the underlying CRS might have influenced this finding. However, upon analysis of all CRS patients we did not observe a divergent expression profile compared to patients who did not exhibit CRS.

We could not identify a distinct CAR T-cell kinetic neither for response to therapy nor for occurrence of CRS or ICANS. This was also the case for any activation or checkpoint molecule. These data point to the existence of multiple pathways or mechanisms involved in anti-tumor activity as well as CRS or ICANS induction, which seems reasonable regarding the rather low likelihood of only one specific marker determining CRS- or ICANS-inducing T-cell subsets. Interestingly, in contrast to CD8^+^ CAR T-cells, we identified an overall decline of CD4^+^ CAR T-cells over time. Importantly, a reduced CAR^+^ T-cell population over time is not necessarily associated with reduced anti-tumor activity as the CAR^+^ T-cell kinetic might also reflect homing towards the CD19^+^ target cells outside of the circulation. While treatment of high-grade CRS typically involves steroids, the majority of CRS patients in our cohort did not require such treatment, suggesting a CRS-treatment unrelated drop in CAR^+^ cells.

Expression kinetics of activation markers revealed highest levels within the CAR T-cell product with a decline over time for the majority of markers. A recent study identified lower expression of PD1 and LAG3 as well as higher levels of the cytotoxicity marker CD107a at peak expansion to be linked with a favorable outcome (19). Due to our limited cohort size and thus not sufficient statistical power, we did not aim to identify a distinct profile associated with response or toxicity. Nevertheless, our longitudinal comparisons allowed for a detailed mapping of expression of various checkpoint molecules relevant in immunotherapy. This could be further enhanced by single cell RNA sequencing, allowing for an in-depth read-out of the transcriptional profile, potentially shedding light on underlying mechanisms of toxicity and efficacy. Therefore, future research should consider inclusion of such methodology in order to correlate outcome parameters with transcriptomics.

However, for all analyses individual patient variables also need to be taken into account, likely influencing outcome as well as potential risk factors for development of CRS/ICANS, e.g. disease status prior to CAR T-cell therapy or concurring inflammation. Considering the restricted statistical power of our small patient cohort, a multivariable analysis to assess the role of these variables was not possible. Instead, we described clinical variables in detail for every patient. Hence, the risk factors can be reviewed in an individual patient approach (**Supplementary Tables S2**, **S3**). Nevertheless, a larger and more diverse validation cohort is required, not only to replicate our findings in an independent patient population, but also to include patients with different CAR T-cell products in order to provide a more comprehensive picture with regard to clinical variables influencing outcome and the development of CRS and ICANS using a multivariable model. Such a validation approach is the prerequisite to test the suitability of the identified T-cell phenotypes as a future biomarker. Ultimately, warranting validation of our results, longitudinal in-depth phenotyping of T-cell subsets in patients undergoing CAR T-cell therapy might help identify patients at risk for adverse events as well as read-out of efficacy of the cell product. Therefore, translation of our findings into clinical practice might lead to improved outcome and safety.

## Data availability statement

The original contributions presented in the study are included in the article/**Supplementary Material**. Further inquiries can be directed to the corresponding authors.

## Ethics statement

The studies involving humans were approved by Ethikkommission Hannover Medical School. The studies were conducted in accordance with the local legislation and institutional requirements. The participants provided their written informed consent to participate in this study. Written informed consent was obtained from the individual(s) for the publication of any potentially identifiable images or data included in this article.

## Author contributions

IO: Conceptualization, Formal analysis, Methodology, Visualization, Writing – original draft. LB: Formal analysis, Methodology, Visualization, Writing – original draft. LR: Formal analysis, Methodology, Writing – review & editing. RS: Formal analysis, Methodology, Validation, Writing – review & editing. JS: Investigation, Writing – review & editing. YX: Writing – review & editing. NM: Investigation, Writing – review & editing. TS: Resources, Writing – review & editing. GB: Investigation, Writing – review & editing. ME: Investigation, Writing – review & editing. AG: Resources, Writing – review & editing. RF: Resources, Writing – review & editing. CS-F: Conceptualization, Formal analysis, Funding acquisition, Investigation, Methodology, Writing – original draft. CK: Conceptualization, Funding acquisition, Investigation, Writing – original draft.
