# Supplementary material for: Brief research report: in-depth immunophenotyping reveals stability of CD19 CAR T-cells over time
Source: Front Immunol. 2024 Jan 22;15:1298598. doi: 10.3389/fimmu.2024.1298598 (PMC10839090; doi:10.3389/fimmu.2024.1298598)
Supplement: Supplementary file 1 [file DataSheet_1.docx]

**Supplementary Material**

**Contents**

**Supplementary Figures**

- - **Supplementary Figure S1.** Overview of patient samples and study workflow.
  - **Supplementary Figure S2.** Integrated analysis of all samples prior to and after infusion.
  - **Supplementary Figure S3.** Detailed phenotyping of clusters from integrated analysis shown in Supplementary Figure S2.

**Supplementary Tables**

- - **Supplementary Table S1.** Antibody panel for spectral flow cytometry.
  - **Supplementary Table S2.** Outcome-stratified cohort characteristics prior to CART therapy.
  - **Supplementary Table S3.** CART infusion product characteristics and response to therapy.
  - **Supplementary Table S4.** CAR T-cell toxicity and treatment.
  - **Supplementary Table S5.** Infections post CART therapy.

# **1. Supplementary Figures**

a

Patients who received Tisagenlecleucel (n=16)

Patients analyzed (n=12)

Patients analyzed (n=12)

Patients analyzed (n=13)

Patients excluded (n=4)

- No samples available (n=2)
- Cell count of CART product not according to manufacturing standard (n=1)
- Insufficient sample quality (n=1)

CART infusion bag samples for analysis (n=11)

- ETP: 2 (2-3)* days post infusion (n=9)
- LTP: 105 (48-130)* days post infusion (n=9)

Patient follow-up samples for analysis

(n=18)

**b**

**Supplementary Figure S1.** Overview of patient samples and study workflow.

a) Patients’ recruitment and samples for analysis. Abbreviations: CART Chimeric antigen receptor T-cell, ETP indicates early time point, LTP late time point. *Median (range).
b) Shown is the study workflow. Sampling was followed by isolation of peripheral blood mononuclear cells. After multi-color staining, spectral flow cytometry was performed. Data were analyzed via conventional 2D gating as well as via an unsupervised cluster analysis, using the Uniform Manifold Approximation and Projection (UMAP) approach for dimensionality reduction (details are described within the Methods section of the main manuscript). ETP indicates early time point and LTP late time point. This figure was created with BioRender.com.

**
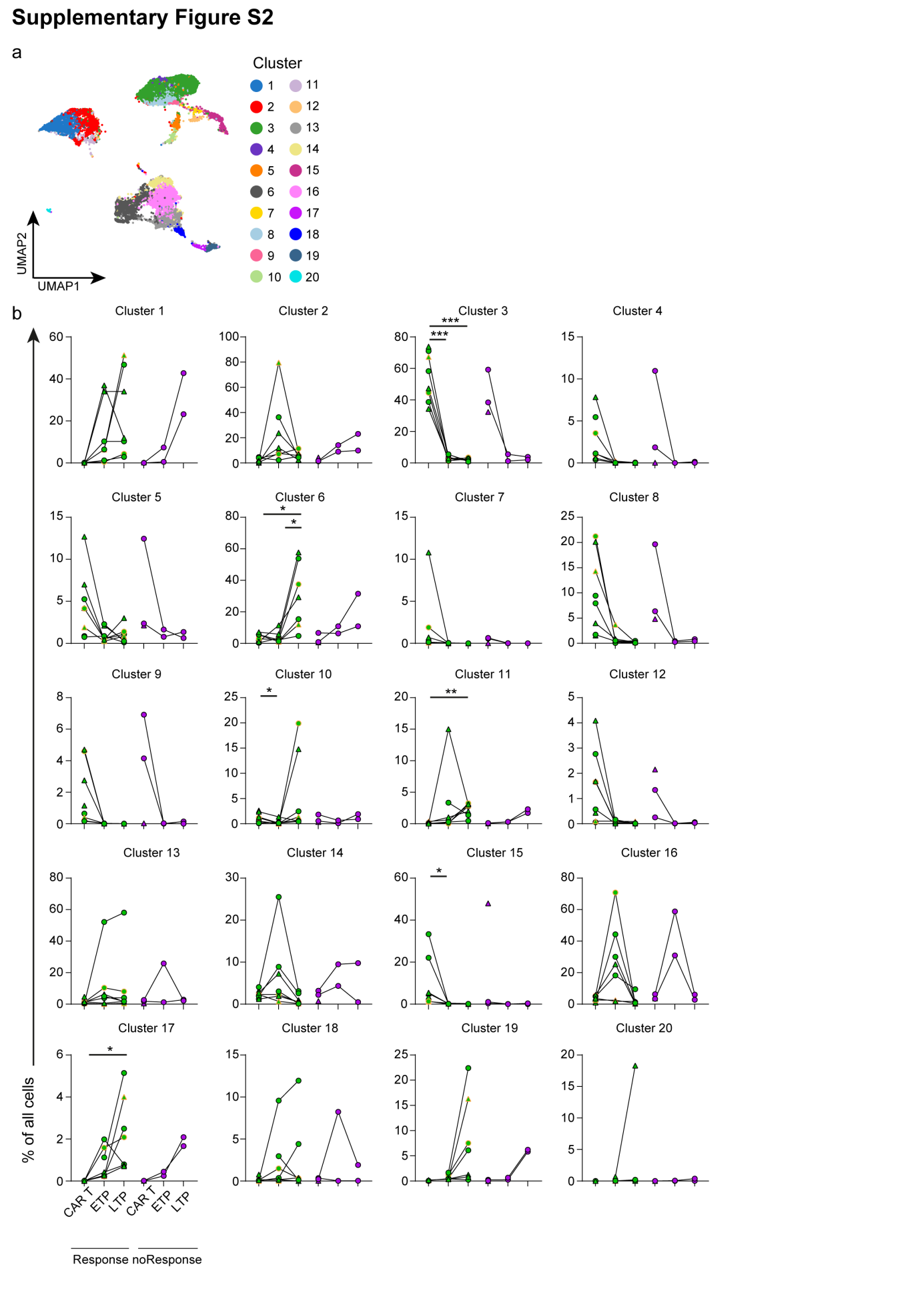
**

**Supplementary Figure S2**. Integrated analysis of all samples prior to and after infusion.

a) UMAP visualization identifying 20 distinct cell clusters of all samples (n=29). b) Paired analysis of cluster frequencies out of all cells from CAR T-cell product (CAR T), early (ETP) and late time point (LTP). Statistics was done by one way ANOVA for paired samples with Tukey’s post hoc test .*p<0.05; **p<0.01; ***p<0.001.

**
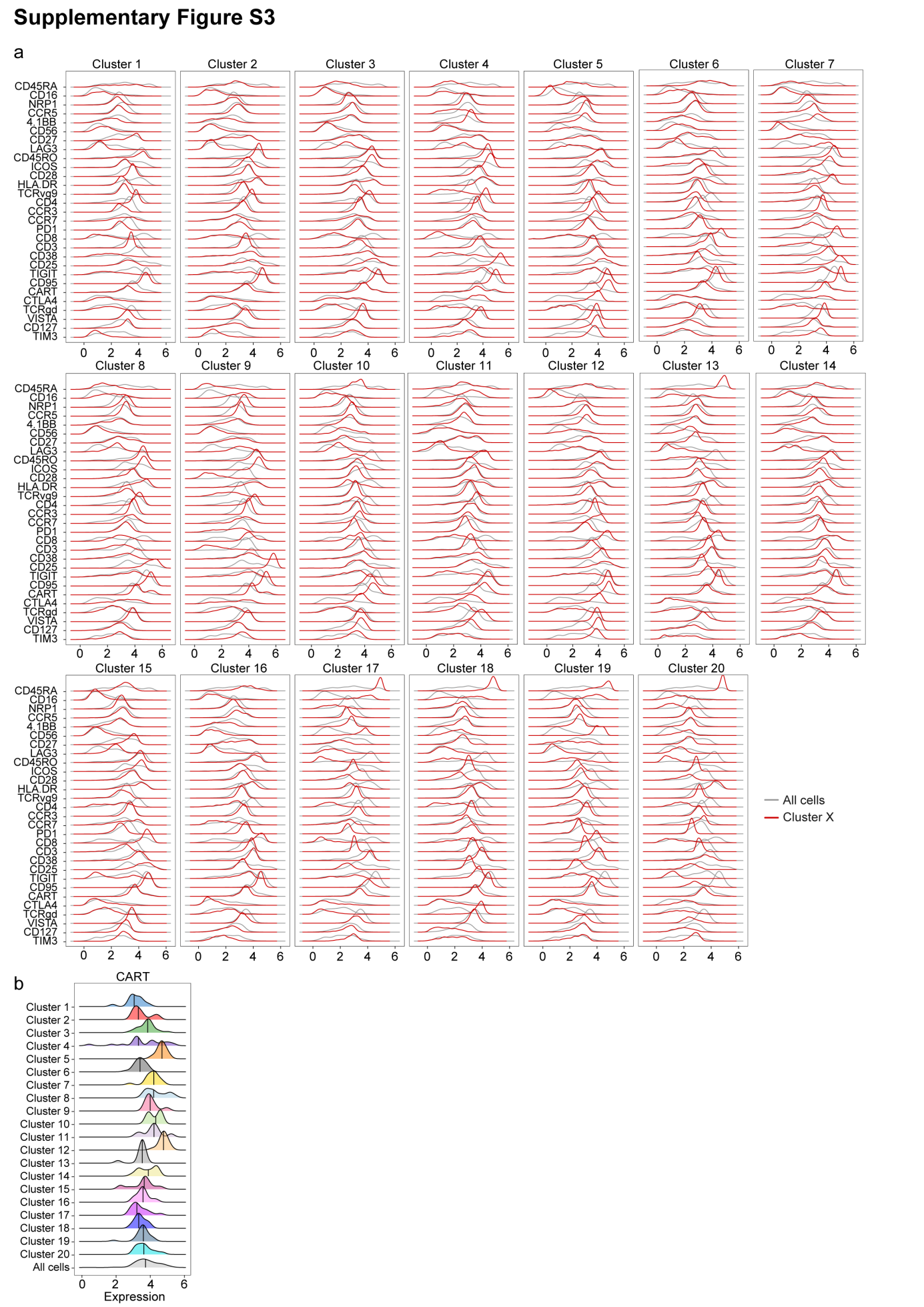
**

**Supplementary Figure S3.** Detailed phenotyping of clusters from integrated analysis shown in Supplementary Figure S2.

a) Ridgeplots showing relative expression of indicated markers across all clusters. Grey lines represent expression across all samples and red lines represent expression in indicated cluster. b) Ridgeplot showing relative expression of CAR^+^ in all clusters.

# **2. Supplementary Tables**

| Specificity | Fluorochrome | RRID | Clone | Lot | Company | Dilution | Purpose |
| --- | --- | --- | --- | --- | --- | --- | --- |
| CD45RA | BUV395 | AB_2740037 | HI100 | 0143332 | BD | 1:100 | Maturity |
| CD16 | BUV496 | AB_2870224 | 3G8 | 9213597 | BD | 1:100 | Monocytes |
| NRP1 | BUV563 | AB_2873486 | U21-1283 | 0295278 | BD | 1:20 | Imm. synapse |
| CCR5 | BUV615 | AB_2875581 | 3A9 | 0323872 | BD | 1:20 | T-helper cells |
| 4-1BB | BUV661 | AB_2871042 | 4B4-1 | 0295062 | BD | 1:20 | Stimulatory IC |
| CD56 | BUV737 | AB_2813880 | NCAM16.2 | 0233660 | BD | 1:20 | NK cells |
| CD27 | BUV805 | AB_2873108 | L128 | 0295263 | BD | 1:20 | Activation |
| LAG3 | BV421 | AB_2629797 | 11C3C65 | B263595 | BioLegend | 1:20 | Inhibitory IC |
| CD45RO | Pacific Blue | AB_493659 | UCHL1 | B274303 | BioLegend | 1:20 | Maturity |
| ICOS | BV480 | AB_2743582 | DX29 | 0295093 | BD | 1:20 | Stimulatory IC |
| CD28 | BV510 | AB_2562030 | CD28.2 | B302905 | BioLegend | 1:20 | Activation |
| HLA-DR | BV570 | AB_2650882 | L243 | B314476 | BioLegend | 1:20 | Activation |
| TCRvg9 | BV605 | AB_2741946 | B3 | 0295071 | BD | 1:20 | γδ T-cells |
| CD4 | Qdot605 | AB_11180611 | S3.5 | 2260863 | Invitrogen | 1:20 | T-helper cells |
| CCR3 | BV711 | AB_2741255 | 5E8 | 0323827 | BD | 1:20 | T-helper cells |
| CCR7 | BV785 | AB_2563630 | G043H7 | B319813 | BioLegend | 1:20 | Differentiation |
| PD1 | FITC | AB_2572163 | NAT105 | B226290 | BioLegend | 1:20 | Inhibitory IC |
| CD3 | AF532 | AB_2819983 | UCHT1 | 2151957 | Invitrogen | 1:100 | T-cells |
| CD8 | SparkBlue550 | AB_11218675 | SK1 | B302398 | BioLegend | 1:100 | CTL |
| CD38 | PerCP-eF710 | AB_1834399 | HB7 | 2044748 | Invitrogen | 1:20 | Activation |
| CD14 | BB700 | AB_2739737 | MΦP9 | 0267076 | BD | 1:20 | Monocytes |
| CD25 | PE | AB_314276 | BC96 | B314333 | BioLegend | 1:20 | Treg |
| TIGIT | PE-eF610 | AB_2723715 | MBSA43 | 2134459 | Invitrogen | 1:20 | Inhibitory IC |
| CD95 | PE-Cy5 | AB_314548 | DX2 | B314186 | BioLegend | 1:20 | Maturity |
| CART | Biotin | AB_2811310 | - | 5191202059 | Miltenyi | 1:20 | CAR T-cells |
| Streptavidin | PE-Cy5.5 | AB_10373840 | - | 2181621 | Invitrogen | 1:20 | CAR T-cells |
| CTLA4 | PE-Cy7 | AB_2563098 | L3D10 | B284847 | BioLegend | 1:20 | Inhibitory IC |
| TCRγδ | APC | AB_2733463 | REA591 | 5201200594 | Miltenyi | 1:50 | γδ T-cells |
| VISTA | AF647 | AB_2744493 | MIH65.rMab | 0167818 | BD | 1:20 | Inhibitory IC |
| CD127 | AF700 | AB_2566200 | A019D5 | B307791 | BioLegend | 1:20 | Treg |
| Viability | Zombie NIR | NA | - | B285746 | BioLegend | 1:400 | Live/dead |
| TIM3 | APC Fire 750 | AB_2632856 | F38-2E2 | B282359 | BioLegend | 1:20 | Inhibitory IC |

**Supplementary Table S1.** Antibody panel for spectral flow cytometry.

Abbreviations: RRID Research Resource Identifiers, Imm. Synapse Immunological synapse, IC Immune checkpoint, CTL Cytotoxic lymphocytes, Treg Regulatory T-cells, CART Chimeric antigen receptor T-cell, NA Not applicable.

|  | All | CR/PR | PD | CRS | ICANS | No CRS/ICANS |
| --- | --- | --- | --- | --- | --- | --- |
| N | 12 | 9 | 3 | 5^#^ | 2^#^ | 6 |
| Age at CART infusion, years | 60 (22-75) | 60 (22-74) | 59 (36-75) | 36 (22-74) | 70 (65-74) | 60 (31-75) |
| Sex, male | n=8 (67%) | n=6 (67%) | n=2 (67%) | n=4 (80%) | n=1 (50%) | n=3 (50%) |
| Disease  DLBCL (%)  tFL (%)  cALL (%) | n=8 (67%)  n=3 (25%)  n=1 (8%) | n=6 (67%)  n=2 (22%)  n=1 (11%) | n=2 (67%)  n=1 (33%)  n=0 (0%) | n=3 (60%)  n=1 (20%)  n=1 (20%) | n=1 (50%)  n=1 (50%)  n=0 (0%) | n=5 (83%)  n=1 (17%)  n=0 (0%) |
| Molecular rearrangements*  Double-hit  Triple-hit | n=4 (36%)  n=2 (18%) | n=2 (25%)  n=2 (25%) | n=2 (67%)  n=0 (0%) | n=1 (25%)  n=1 (25%) | n=1 (50%)  n=0 (0%) | n=2 (33%)  n=1 (17%) |
| Ann Arbor Stage ≥3  at 1^st^ diagnosis* | n= 9 (82%) | n=6 (75%) | n=3 (100%) | n=3 (75%) | n=2 (100%) | n=5 (83%) |
| Extranodal sites at 1^st^ diagnosis* | n=10 (91%) | n=7 (88%) | n=3 (100%) | n=3 (75%) | n=1 (50%) | n=6 (100%) |
| IPI score ≤2 at 1^st^ diagnosis* | n=8 (73%) | n=5 (63%) | n=3 (100%) | n=3 (75%) | n=1 (50%) | n=5 (83%) |
| Previous lines of antineoplastic therapy | 3 (2-8) | 3 (2-8) | 3 (3-4) | 3 (2-8) | 3 (2-4) | 3 (2-4) |
| Autologous SCT* | n=6 (55%) | n=5 (63%) | n=1 (33%) | n=2 (50%) | n=1 (50%) | n=4 (67%) |
| Ann Arbor Stage ≥3  prior to CART* | n=10 (91%) | n=7 (88%) | n=3 (100%) | n=4 (100%) | n=2 (100%) | n=5 (83%) |
| Elevated LDH prior to CART | n=5 (42%) | n=2 (22%) | n=3 (100%) | n=2 (40%) | n=0 (0%) | n=3 (50%) |
| Extranodal sites prior to CART* | n=10 (91%) | n=7 (88%) | n=3 (100%) | n=3 (75%) | n=1 (50%) | n=6 (100%) |
| ECOG ≤1 prior to CART | n=10 (83%) | n=8 (89%) | n=2 (67%) | n=4 (80%) | n=2 (100%) | n=5 (83%) |
| PD prior to CART | n=12 (100%) | n=9 (100%) | n=3 (100%) | n=5 (100%) | n=2 (100%) | n=6 (100%) |
| Patients who received standard lymphodepleting therapy | n=10 (83%) | n=7 (78%) | n=3 (100%) | n=4 (80%) | n=1 (50%) | n=6 (100%) |
| Bridging therapy  Bridging CTx  Bridging RTx | n=6 (50%)  n=2 (17%) | n=4 (44%)  n=1 (11%) | n=2 (67%)  n=1 (33%) | n=3 (60%)  n=1 (20%) | n=1 (50%)  n=0 (0%) | n=3 (50%)  n=1 (17%) |
| Time from apheresis to infusion, days | 61 (35-196) | 70 (35-196) | 56 (38-60) | 62 (35-115) | 80 (70-89) | 57 (38-196) |

**Supplementary Table S2.** Outcome-stratified cohort characteristics prior to CART therapy.

For continuous variables the median (range) and for categorical variables the absolute number (%) is shown.

Abbreviations: CRS Cytokine Release Syndrome, ICANS Immune effector-cell associated neurotoxicity syndrome, DLBCL Diffuse large B-cell lymphoma, tFL Transformed follicular lymphoma, cALL Common acute lymphoblastic leukemia, IPI International prognostic index, SCT Stem cell transplantation, ECOG European Cooperative Oncology Group Status Scale, PD Progressive disease, CTx Chemotherapy, Rtx Radiation therapy.

* Not applicable for patient 20/01 due to common acute lymphoblastic leukemia as underlying disease.

^#^ Patient 19/07 had concurrent CRS and ICANS, therefore she is listed in both columns.

| Pat ID | Cell  viability  in % | Total  cell count  x10^9^ | Calculated  dose x10^8^ | Response  ~30 days  post infusion | Response  ~90 days  post infusion | PFS in mo | OS in mo | Alive 24 months post CART infusion |
| --- | --- | --- | --- | --- | --- | --- | --- | --- |
| 19/03 | 95,1 | 1,08 | 2,0046 | CR | CR | 14 | 14 | Yes |
| 19/04 | 83,2 | 1,34 | 2,5804 | NR | NR | 0 | 0 | No |
| 19/06 | 92,2 | 1,54 | 1,7132 | PR | Relapse | 3 | 29 | Yes (alloSCT 6mo post infusion) |
| 19/07 | 96 | 3,81 | 3,4 | PR | Relapse | 3 | 5 | No |
| 19/08 | 96,9 | 1,64 | 3,5 | NR | NR | 0 | 0 | No |
| 19/09 | 93,3 | 1,43 | 1,0149 | NR | NR | 0 | 8 | No |
| 19/10 | 87,8 | 2,24 | 3,2 | PR | Relapse | 6 | 21 | No |
| 19/11 | 94,4 | 1,89 | 2,8871 | PR | CR | 8 | 31 | Yes |
| 19/12 | 93 | 1,99 | 3,2 | PR | NR | 1 | 6 | No |
| 20/01 | 79,2 | 1,17 | 0,02 | CR | CR | 24 | 25 | Yes |
| 20/04 | 91,5 | 3,49 | 2,1286 | PR | CR | 13 | 17 | Yes |
| 20/05 | 79,9 | 1,63 | 2,3617 | NR | NR | 0 | 2 | No |

**Supplementary Table S3.** CART infusion product characteristics and response to therapy.

Abbreviations: Pat ID Patient ID, PFS Progression-free survival, mo Months, OS Overall survival, CR Complete response, PR Partial response, NR No response, CART Chimeric antigen receptor T-cell, alloSCT Allogeneic stem cell transplantation.

| Pat ID | Hospitalization (days) | ICU care (days) | CRS grade | CRS treatment | ICANS grade | ICANS treatment |
| --- | --- | --- | --- | --- | --- | --- |
| 19/03 | 9 | 0 | 0 | - | 0 | - |
| 19/04 | 23 | 17 | 4 | Toc, Dex, Mp, VP, MV, RRT, ECA, PP | 0 | - |
| 19/06 | 13 | 0 | 0 | - | 0 | - |
| 19/07 | 12 | 8 | 2 | Toc | 2 | Dex |
| 19/08 | 5 | 0 | 1 | None | 0 | - |
| 19/09 | 10 | 0 | 0 | - | 0 | - |
| 19/10 | 18 | 0 | 0 | - | 2 | Dex |
| 19/11 | 12 | 0 | 0 | - | 0 | - |
| 19/12 | 10 | 0 | 1 | None | 0 | - |
| 20/01 | 15 | 0 | 1 | None | 0 | - |
| 20/04 | 15 | 0 | 0 | - | 0 | - |
| 20/05 | 14 | 0 | 0 | - | 0 | - |

**Supplementary Table S4.** CAR T-cell toxicity and treatment.

Abbreviations: Pat ID Patient ID, ICU Intensive care unit, CRS Cytokine release syndrome, ICANS Immune effector cell-associated neurotoxicity syndrome, Dex Dexamethasone, ECA Extracoporeal cytokine adsorption, Mp Methylprednsiolone, PP Plasmapheresis, RRT Renal replacement therapy, VP Vasopressors.

| Pat ID | Diagnosis | Germ | Time post CART infusion | Treatment |
| --- | --- | --- | --- | --- |
| 19/03 | Inguinal rash | Enterococcus species, Candida albicans | 6 days | Not specified* |
| 19/04 | Cytomegalovirus reactivation  Sepsis | Cytomegalovirus  Enterococcus faecium | 14 days  15 days | Ganciclovir, Foscavir  Meropenem, Linezolid |
| 19/06 | - | - | - | - |
| 19/07 | Fungal pulmonary infection | Not specified | 1 month | Voriconazole |
| 19/08 | - | - | - | - |
| 19/09 | - | - | - | - |
| 19/10 | Thrush  Urosepsis | -  3MDR Escherichia coli | 14 days  2 months | Fluconazole  Meropenem |
| 19/11 | Pneumonia | - | 3 months | Not specified* |
| 19/12 | - | - | - | - |
| 20/01 | - | - | - | - |
| 20/04 | Urinary tract infection  Port-site infection | Escherichia coli  Staphylococcus epidermidis | 1 month  2 months | Not specified  Clindamycin |
| 20/05 | Bacteremia | Staphylococcus epidermidis | 6 days | Piperacillin-Tazobactam, Vancomycin, Meropenem |

**Supplementary Table S5.** Infections post CART therapy.

Abbreviations: Pat ID Patient ID, 3MDR Multi-drug resistant Escherichia coli.

* Treatments were administered by external medical care providers.
